# The Cytosolic Protein G0S2 Maintains Quiescence in Hematopoietic Stem Cells

**DOI:** 10.1371/journal.pone.0038280

**Published:** 2012-05-31

**Authors:** Takeshi Yamada, Chun Shik Park, Audrea Burns, Daisuke Nakada, H. Daniel Lacorazza

**Affiliations:** 1 Department of Pathology & Immunology, Baylor College of Medicine, Texas Children's Hospital, Houston, Texas, United States of America; 2 Department of Molecular and Human Genetics, Baylor College of Medicine, Texas Children's Hospital, Houston, Texas, United States of America; 3 Department of Pediatrics, Baylor College of Medicine, Texas Children's Hospital, Houston, Texas, United States of America; French Blood Institute, France

## Abstract

Bone marrow hematopoietic stem cells (HSCs) balance proliferation and differentiation by integrating complex transcriptional and post-translational mechanisms regulated by cell intrinsic and extrinsic factors. We found that transcripts of G_0_/G_1_ switch gene 2 (G0S2) are enriched in lineage^−^ Sca-1^+^ c-kit^+^ (LSK) CD150^+^ CD48^−^ CD41^−^ cells, a population highly enriched for quiescent HSCs, whereas G0S2 expression is suppressed in dividing LSK CD150^+^ CD48^−^ cells. Gain-of-function analyses using retroviral expression vectors in bone marrow cells showed that G0S2 localizes to the mitochondria, endoplasmic reticulum, and early endosomes in hematopoietic cells. Co-transplantation of bone marrow cells transduced with the control or G0S2 retrovirus led to increased chimerism of G0S2-overexpressing cells in femurs, although their contribution to the blood was reduced. This finding was correlated with increased quiescence in G0S2-overexpressing HSCs (LSK CD150^+^ CD48^−^) and progenitor cells (LS^−^K). Conversely, silencing of endogenous G0S2 expression in bone marrow cells increased blood chimerism upon transplantation and promoted HSC cell division, supporting an inhibitory role for G0S2 in HSC proliferation. A proteomic study revealed that the hydrophobic domain of G0S2 interacts with a domain of nucleolin that is rich in arginine-glycine-glycine repeats, which results in the retention of nucleolin in the cytosol. We showed that this cytosolic retention of nucleolin occurs in resting, but not proliferating, wild-type LSK CD150^+^ CD48^−^ cells. Collectively, we propose a novel model of HSC quiescence in which elevated G0S2 expression can sequester nucleolin in the cytosol, precluding its pro-proliferation functions in the nucleolus.

## Introduction

Over an individual's lifetime, the long-lived hematopoietic stem cells (HSCs) are confronted with a number of different potential fates: maintenance of the HSC pool (self-renewal), production of blood cells on demand (differentiation), mobilization, death, or entry into a reversible cell cycle arrest in which they remain poised to re-enter cell division and differentiation (quiescence). A balanced regulation of these processes ensures a continuous supply of hematopoietic cells without leading to stem cell exhaustion or bone marrow (BM) failure. The quiescent state preserves the stemness of HSCs as well as their ability to efficiently reconstitute ablated hosts upon transplantation. An emerging paradigm suggests that quiescence is controlled by cell intrinsic factors (i.e., Bmi1, Mel18, Mll, ELF4, and c-myb) in addition to microenvironmental cues [1], [2], [3]. Despite its critical role in hematopoiesis, the molecular regulation of quiescence remains a poorly understood process, particularly at the post-transcriptional level [4], [5]. Thus, a better understanding of the regulatory mechanisms that control the proliferation and differentiation of HSCs will aid in the development of new approaches to accelerate hematologic recovery from treatment-induced cytopenia.

G0S2 is a basic protein with an ill-defined function that was first identified in lectin-activated lymphocytes [6]. It has been postulated that G0S2 regulates the G_0_/G_1_ phase of the cell cycle by either releasing lymphocytes from quiescence (G_0_ to G_1_ transition) or by promoting proliferation (G_1_ to S phase transition) [6], [7]. Several reports have suggested that G0S2 is a multifaceted protein with disparate functions related to proliferation, metabolism, inflammation, and carcinogenesis. G0S2 induces the differentiation of 3T3-L1 fibroblasts into adipocytes downstream of the peroxisome-proliferator-activated receptor (PPAR) and inhibits lipolysis by interacting with adipose triglyceride lipase [8], [9], [10]. The fact that the *G0S2* gene is epigenetically silenced in head and neck cancers, squamous lung cancer, and cisplatin-resistant cancer cells suggests a role in tumor formation and chemoresistance [11], [12]. However, transcriptome analyses showed that G0S2 expression is elevated in endometriosis [13], bronchial epithelial cells treated with retinoic acid [14], senescent dermal fibroblasts [15], BM cells from patients with rheumatoid arthritis [16], and peripheral mononuclear cells from patients with vasculitis and psoriasis [17], [18]. Interestingly, rheumatoid arthritis and psoriasis patients displayed a low frequency of CD34-positive cells in the peripheral blood and low counts of colony-forming cells with high proliferative potential in the BM [19], [20]. Although the molecular basis of these findings has not yet been elucidated, they suggest that elevated levels of G0S2 may correlate with inefficient hematopoiesis.

Nucleolin is a multifunctional protein that is predominantly localized to the nucleolus but is also detected in the nucleoplasm and cytosol and at the cell surface [21]. Nucleolin indirectly promotes cell growth by regulating the transcription of ribosomal DNA in the nucleolus, maturation of pre-ribosomal RNA in the nucleus, and transport of ribonucleoproteins and ribosomal particles to the cytosol for final assembly [22]. In addition, nucleolin stabilizes mRNA, enhances translation, and shuttles proteins into the nucleus [23], [24], [25]. Nucleolin's ability to increase protein biosynthesis and cell mass suggests that this protein may also help to control the cell cycle. In fact, rapidly dividing cancer cells show increased nucleolin expression [26].

The role of G0S2 in the proliferation of hematopoietic cells has not been investigated since its identification in lymphocytes [6]. In this study, we show that ectopic expression of G0S2 increases the percentage of lineage^−^ Sca-1^+^ c-kit^+^ CD150^+^ CD48^−^ cells in the G_0_ phase of the cell cycle and reduces blood chimerism in competitive transplantation assays. We identified G0S2 protein partners and found that the hydrophobic domain of G0S2 interacts with the arginine-glycine-glycine (RGG)-rich domain of nucleolin, resulting in the cytosolic retention of nucleolin and reduced proliferation of HSCs. We propose a new model in which HSC quiescence is mediated by the elevated expression of G0S2 and sequestration of nucleolin in the cytosol.

## Results

### G0S2 is expressed in hematopoietic stem cells

**Figure 1:**
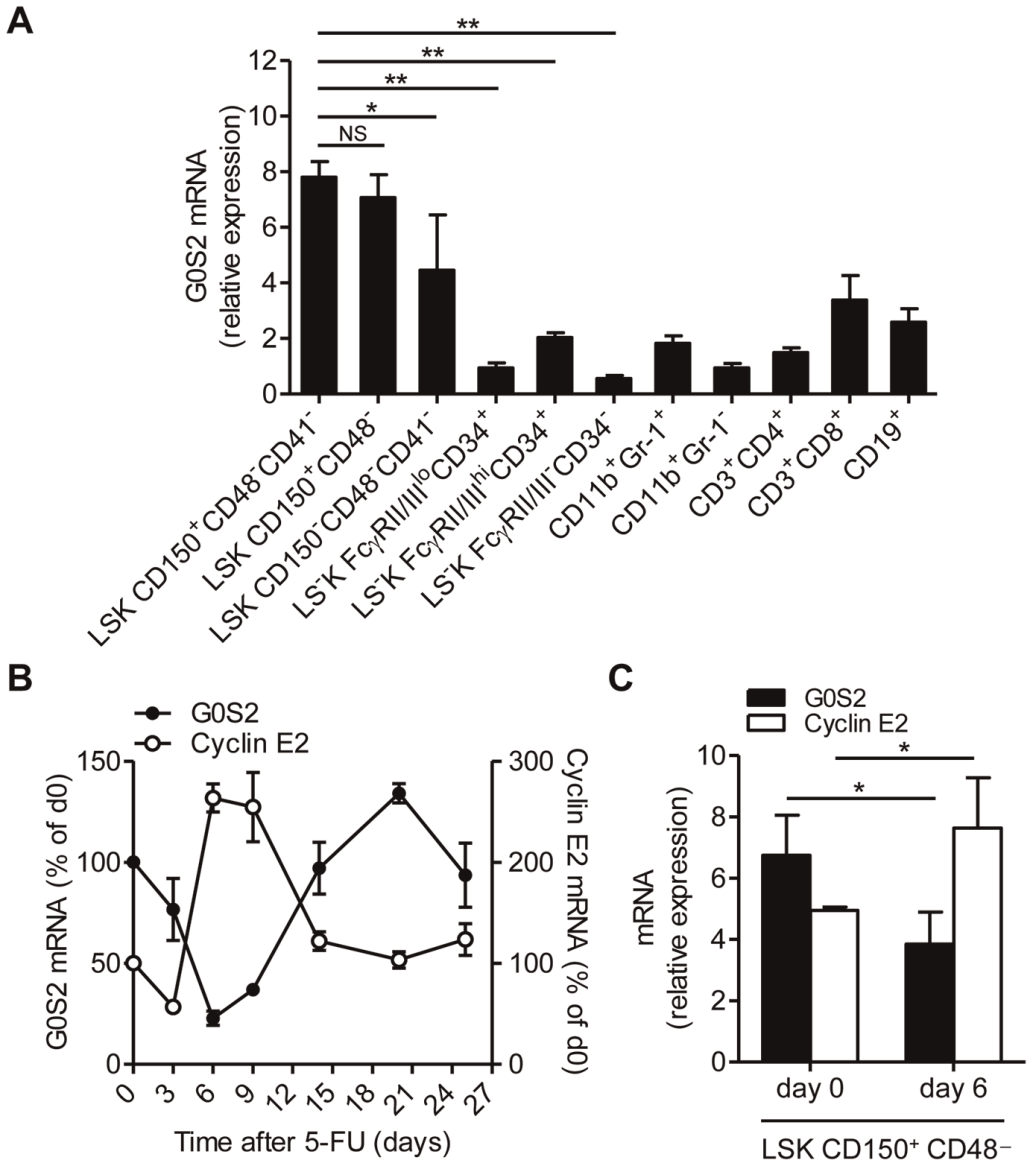
G0S2 is expressed in dormant hematopoietic stem cells. (A) G0S2 transcripts were measured by quantitative real-time PCR in bone marrow hematopoietic stem and progenitor cells based on SLAM markers and mature myeloid and lymphoid cells purified from the spleen (*n* = 3). Statistical significance is indicated between HSCs and progenitor cells (MPP, CMP, GMP, MEP). (B) Expression of G0S2 and cyclin E2 in BM cells isolated at different times after administration of a single dose of 5-FU in C57BL/6 mice. The relative expression levels of G0S2 and cyclin E2 are shown as percentages of basal levels (*n* = 3–4). (C) Transcript levels of G0S2 and cyclin E2 were measured in LSK CD150^+^ CD48^−^ cells purified from untreated or 5-FU-treated (day 6) mice (*n* = 3). The data represent the mean and standard deviation of each experiment. *, *P*<0.05 and **, *P*<0.01 (two-tailed Student's *t*-test).

We first measured G0S2 expression in purified HSCs, progenitor cells, and mature cells by quantitative real-time PCR to gain insight into its function in bone marrow hematopoiesis. G0S2 transcripts are enriched in two HSC populations: lineage^−^ Sca-1^+^ c-kit^+^ (LSK) CD150^+^ CD48^−^ CD41^−^ (LSK CD150^+^ CD48^−^ CD41^−^) and LSK CD150^+^ CD48^−^ cells (Figure 1A). Interestingly, low G0S2 expression was detected in MPP (LSK CD150^−^ CD48^−^ CD41^−^), CMP (LS^−^K FcγRII/III^lo^ CD34^+^), GMP (LS^−^K FcγRII/III^hi^ CD34^+^), MEP (LS^−^K FcγRII/III^−^ CD34^−^), and mature myeloid and lymphoid cells (Figure 1A). These findings suggested that G0S2 might play a role in the maintenance of HSCs.

Because *G0S2* is a G_0_/G_1_ switch gene, we induced HSC proliferation *in vivo* by administering a single dose of 5-fluorouracil (5-FU) to wild-type mice and then quantified G0S2 expression in BM cells at different times during regenerative proliferation to determine whether G0S2 regulates proliferation. Similar to BM cellularity and in contrast to the expression of cyclin E2, G0S2 expression was rapidly downregulated in BM cells, with a nadir occurring at day 6 after 5-FU injection (Figure 1B). This suppression of G0S2 expression in proliferating hematopoietic cells suggests an inhibitory role in cell division. To determine whether this expression pattern could be attributed to HSCs, we measured cyclin E2 and G0S2 expression in LSK CD150^+^ CD48^−^ cells purified from untreated (day 0) or 5-FU-treated (day 6) mice. As predicted, G0S2 expression was inversely correlated with cyclin E2 expression in proliferating LSK CD150^+^ CD48^−^ cells (Figure 1C). Because LSK CD150^+^ CD48^−^ cells have been used by several groups to immunophenotypically define HSCs with long-term repopulating potential [27], [28], [29], [30], these data suggest that G0S2 has an inhibitory effect on HSC proliferation, and its expression is therefore suppressed during regenerative hematopoiesis.

### Ectopic G0S2 expression reduces the contribution of HSCs to the peripheral blood

**Figure 2:**
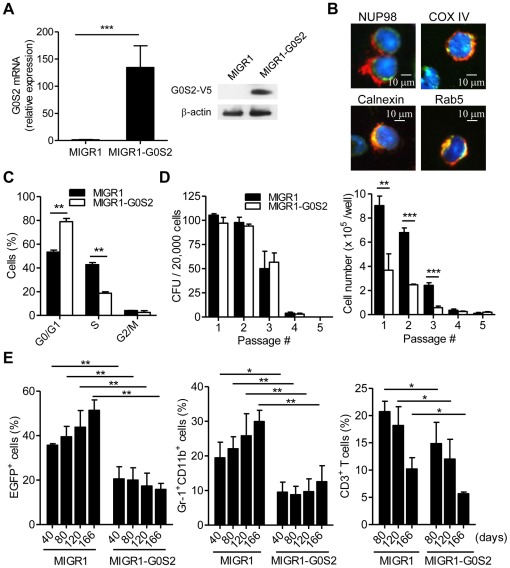
Ectopic G0S2 expression reduces hematological reconstitution after BM transplantation. BM cells were transduced with control MIGR1 or MIGR1-G0S2 (V5-tagged) retroviruses to study the effect of G0S2 on hematopoiesis. (A) Retroviral expression of G0S2 was measured by quantitative real-time PCR and immunoblotting (*n* = 3). (B) Transduced BM cells were used to study subcellular localization using anti-V5 (red), DAPI (blue) and anti-Nup98, COX IV, Calnexin or Rab5 (green) antibodies. The data represent three independent experiments. (C) The cell-cycle distribution of transduced BM cells was analyzed using nuclear staining with propidium iodine and flow cytometry (*n* = 3). (D) Transduced BM cells were transplanted into lethally irradiated mice and the frequency (CFU) and colony cell number were enumerated in methylcellulose culture (*n* = 3) after three months of hematologic reconstitution. (E) The contribution of donor-derived cells to myeloid and T cell populations in the peripheral blood was analyzed after transplantation by flow cytometry at different times post-transplant (*n* = 3–4). The data are representative of two independent experiments. *, *P*<0.05, **, *P*<0.01, and ***, *P*<0.001 (two-tailed Student's *t*-test).

We decided to study the effects of G0S2 in both *in vitro* and *in vivo* hematopoiesis using a gain-of-function mouse model because G0S2 was found to be upregulated in hematopoietic cells from patients suffering from inflammatory disorders and presenting low frequencies of primitive hematopoietic progenitor cells [19], [20]. BM cells from 5-FU-treated C57BL/6 (B6) mice were transduced with either MIGR1 V5-tagged G0S2 or empty MIGR1 retrovirus. Ectopic G0S2 expression was confirmed in BM cells positive for enhanced green fluorescent protein (EGFP) by quantitative real-time PCR and immunoblotting (Figure 2A). We investigated the subcellular localization of G0S2 in transduced BM cells by immunofluorescence using anti-V5 antibodies and a panel of antibodies that label different organelles. This analysis revealed that G0S2 localizes to the area adjacent to the nuclear envelope (Nup98) and also colocalizes with COX IV (mitochondria), calnexin (endoplasmic reticulum) and Rab5 (early endosomes) (Figure 2B). These findings are in accord with previous reports indicating that G0S2 localizes to the endoplasmic reticulum and mitochondria of 3T3-L1 and HeLa cells [8], [31]. A distribution among multiple cytosolic organelles suggests that G0S2 function in hematopoietic cells may depend on its spatial expression pattern.

The difference in G0S2 expression between resting and activated LSK CD150^+^ CD48^−^ cells indicates that G0S2 may control proliferation in primitive hematopoietic progenitor cells. An analysis of DNA content in BM cells transduced with MIGR1 or MIGR1-G0S2 revealed that G0S2 expression reduced the percentage of BM cells in S phase (Figure 2C). Following BM transplant, EGFP-positive BM cells were plated in methylcellulose cultures to assess the capacity of hematopoietic progenitor cells to generate myeloid colonies. No significant differences were observed in the number of colony-forming units (CFUs) in G0S2-overexpressing BM cells serially replated on methylcellulose, indicating that forced G0S2 expression did not alter the initial number of colony-forming cells and proliferation of more primitive hematopoietic stem/progenitor cells (Figure 2D). However, the number of cells recovered from each passage was significantly lower in the G0S2-expressing BM cells because of a smaller size of the colonies (Figure 2D), indicating that ectopic G0S2 expression reduces the proliferation of colony-forming myelopoietic progenitor cells. The use of cytokines in the methylcellulose cultures with strong pro-proliferative function could overshadow differences in the proliferation at steady state.

Alterations in the proliferative capacity of G0S2-overexpressing BM cells may affect their capacity to reconstitute ablated recipient mice. We therefore transplanted BM cells transduced with either the control or the V5-tagged G0S2 retrovirus (60% EGFP-positive cells) into lethally irradiated mice to test the long-term hematopoietic reconstitution of multiple lineages. Donor-derived blood cells were monitored in the peripheral blood by flow cytometry for up to 6 months. Transplantation of G0S2-overexpressing BM cells led to reduced blood chimerism, but the donor cells did contribute to blood cell populations over the long-term (Figure 2E). In addition, we found significant reductions in granulocytes (Gr-1^+^ CD11b^+^) and T cells (CD3^+^) in mice transplanted with G0S2-overexpressing BM cells (Figure 2E). The lower contribution of G0S2-overexpressing BM cells to peripheral blood observed more than 5 months post transplantation can likely be attributed to a lower proliferative capacity of hematopoietic stem and progenitor cells. To ensure that observed effects were due to sustained G0S2 expression, we confirmed the expression by immunoblotting in blood cells from chimeric mice 6 months after transplantation (not shown). We also ruled out the possibility that G0S2 overexpression caused cell death in BM cells (Figure S1).

**Figure 3:**
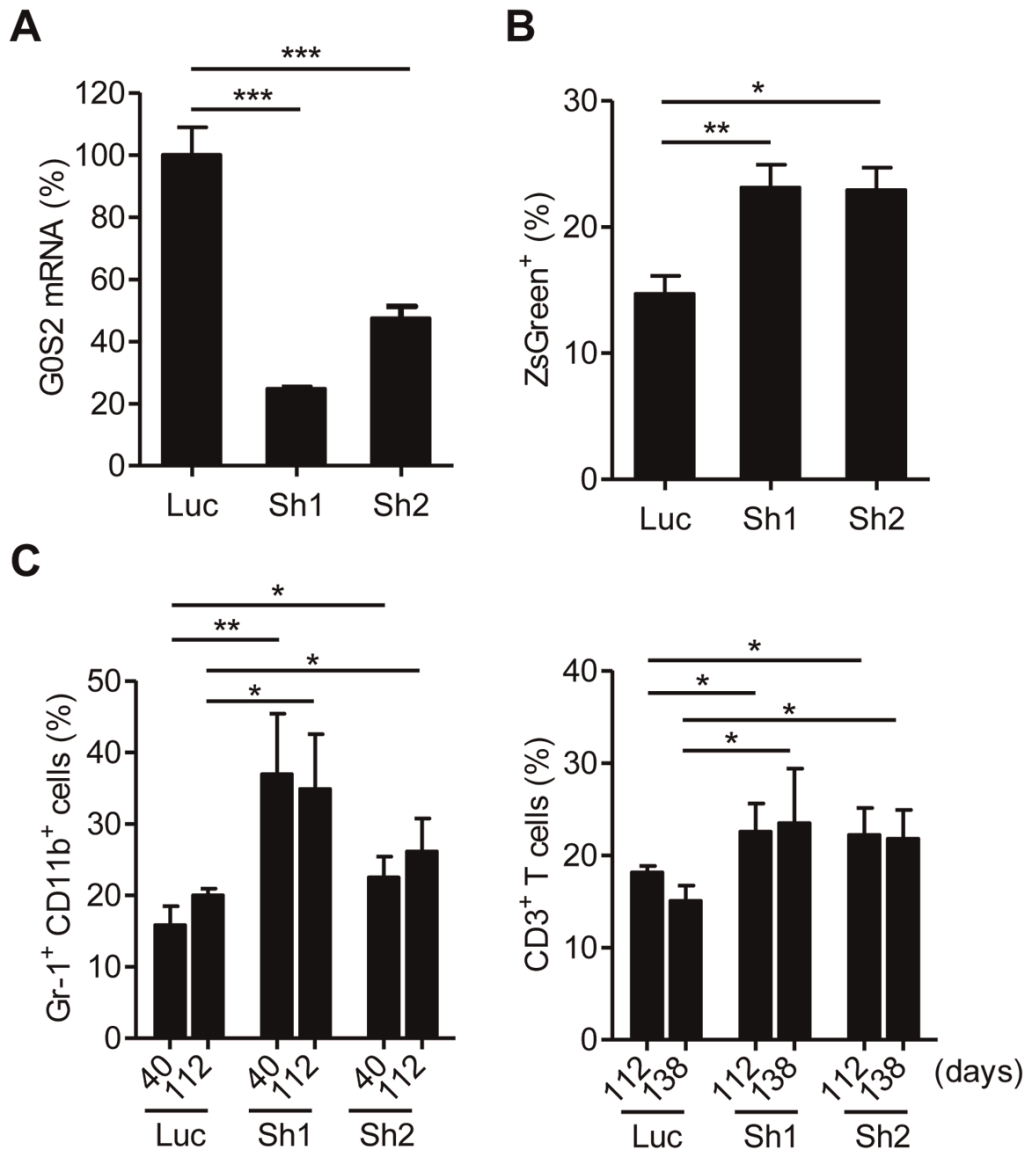
Silencing of endogenous G0S2 expression in BM cells increases blood chimerism upon transplantation. Expression of the endogenous G0S2 gene was silenced in BM cells using two G0S2-specific pSIREN-shRNA retroviruses (Sh1, Sh2). Luciferase pSIREN-shRNA (Luc) was used as a control. (A) Knockdown efficiency was determined by quantitative real-time PCR in transduced BM cells (*n* = 4). (B) Sixteen weeks after transplantation, blood chimerism was measured by flow cytometry (*n* = 3–4). (C) The contribution of donor-derived cells to the Gr-1^+^ CD11b^+^ and CD3^+^ T cell populations was analyzed at different times after the transplant (*n* = 3–4). *, *P*<0.05, **, *P*<0.01, and ***, *P*<0.001 (two-tailed Student's *t*-test).

Although retroviral expression of G0S2 did not cause significant apoptosis in hematopoietic cells, high levels of expression may have nonspecifically altered cell proliferation and differentiation. Therefore, it was important to also determine the effect of G0S2 loss-of-function in HSCs. We chose an shRNA-mediated method to silence endogenous G0S2 expression in BM cells because mice with a homozygous deletion of the *G0S2* gene are not yet available. Two G0S2-specific shRNA pSIREN retroviruses (Sh1 and Sh2) were used to silence G0S2. A luciferase shRNA retrovirus (Luc) was used as a control. The G0S2-specific shRNA retroviruses reduced endogenous G0S2 expression in BM cells by 80% and 60% (Sh1 and Sh2, respectively) (Figure 3A). In contrast to the overexpression results, G0S2 silencing increased the capacity of HSCs to contribute to peripheral blood (Figure 3B). We also observed that the extent of silencing was correlated with the increase in percentages of Gr1^+^ CD11b^+^ and CD3^+^ T cells, particularly short-lived granulocytes, suggesting a dosage effect in stem cell function (Figure 3C).

**Figure 4:**
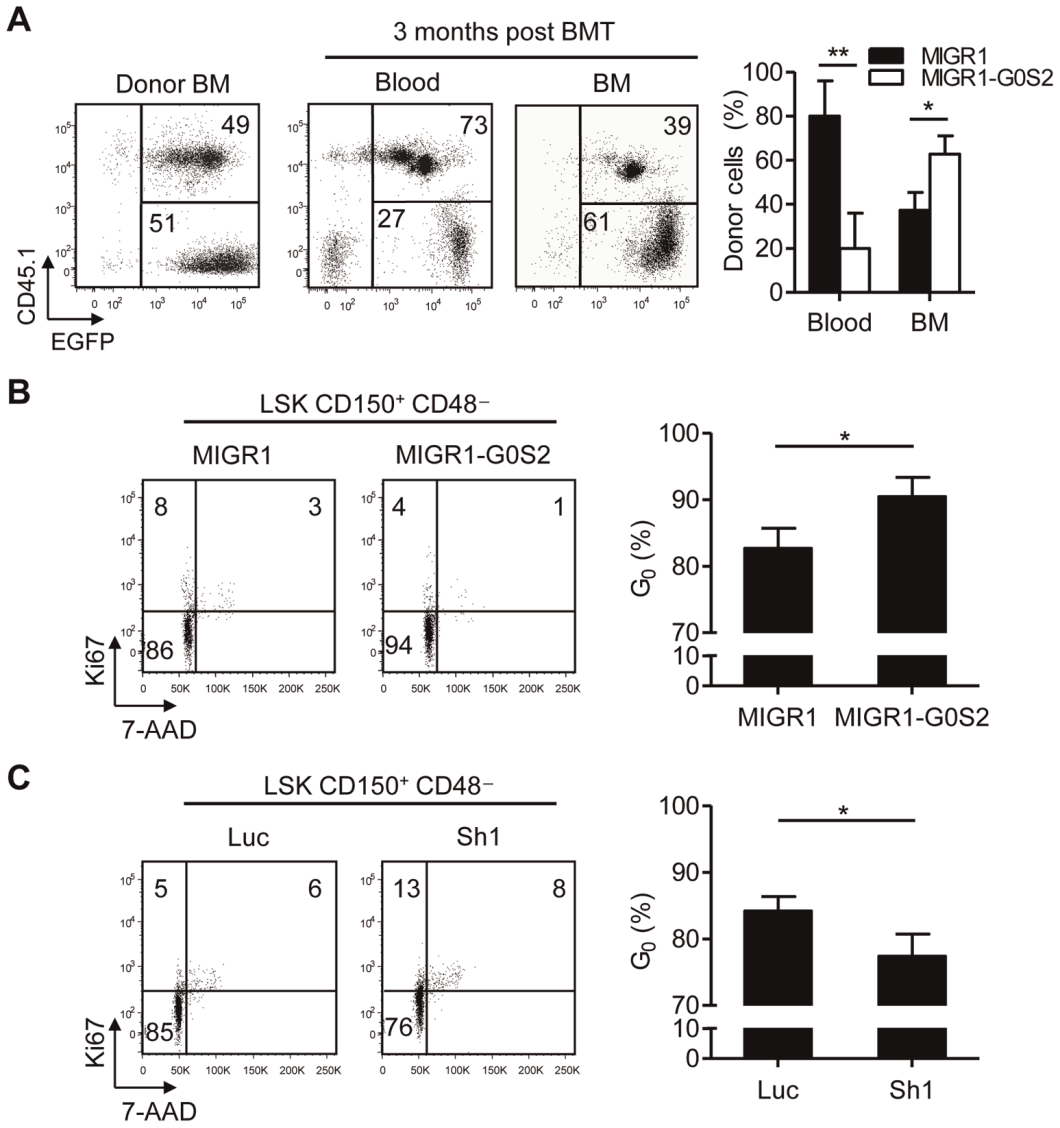
G0S2 enhances the quiescence of hematopoietic stem cells. (A) To analyze the reconstitution potential of G0S2-overexpressing BM cells relative to wild-type BM cells, we performed a competitive transplantation with a mixture of BM cells transduced with the MIGR1 or MIGR1-G0S2 retrovirus (*n* = 5). EGFP^+^ CD45.1^+^ and EGFP^+^ CD45.1^−^ cells were derived from MIGR1 and MIGR1-G0S2 BM cells, respectively. The data represent two independent experiments. (B) The role of G0S2 in HSC quiescence was examined using a gain-of-function model. Flow cytometric analyses of Ki67 and 7-AAD were performed in LSK CD150^+^ CD48^−^ cells purified from chimeric mice transplanted with cells containing the control or G0S2 retrovirus (*n* = 3–4). Quiescent HSCs were defined as Ki67-negative cells (G_0_) with a 2n DNA content. (C) Flow cytometric analyses of Ki67 and 7-AAD were performed in LSK CD150^+^ CD48^−^ cells purified from mice transplanted with G0S2-shRNA or the control retrovirus (*n* = 4–5). *, *P*<0.05 and **, *P*<0.01 (two-tailed Student's *t*-test).

We further examined the effect of G0S2 overexpression on HSCs by transplanting a 1∶1 mixture of B6.SJL BM cells (CD45.1^+^) transduced with the MIGR1 retrovirus and B6 BM cells (CD45.1^−^) transduced with the MIGR1-G0S2 retrovirus into B6 mice. Three months later, CD45.1 and EGFP expression levels were monitored in the peripheral blood and BM by flow cytometry to distinguish blood cells derived from control HSCs (MIGR1, CD45.1^+^ EGFP^+^) and G0S2-overexpressing HSCs (MIGR1-G0S2, CD45.1^−^ EGFP^+^). Surprisingly, the vast majority of blood cells were derived from control HSCs, despite a clear predominance of G0S2-overexpressing cells in the BM (Figure 4A). This result might be attributed to a combination of increased homing to the BM upon transplantation (Figure S1) and lower steady-state contributions to the blood caused by reduced proliferation and/or differentiation.

### G0S2 promotes quiescence in hematopoietic stem cells

To directly assess whether G0S2 inhibits HSC proliferation, we purified LSK CD150^+^ CD48^−^ cells from chimeric mice transplanted with either MIGR1-G0S2- or pSIREN-shG0S2-transduced BM cells. Cell cycle parameters were then determined by dual flow cytometric detection of Ki67 and 7-AAD, markers of proliferation and DNA content, respectively. LSK CD150^+^ CD48^−^ cells negative for Ki67 with a 2n DNA content were defined as ‘quiescent’ HSCs (G_0_ phase of the cell cycle). Approximately 85% of wild-type LSK CD150^+^ CD48^−^ cells were in the G_0_ phase of the cell cycle, emphasizing that this population is highly enriched for dormant HSCs. Interestingly, ectopic expression of G0S2 further increased the quiescence of LSK CD150^+^ CD48^−^ cells (Figure 4B). As shown in Fig. 1, LSK CD150^+^ CD48^−^ cells already express high levels of G0S2; therefore, the increase in HSC quiescence observed upon G0S2 overexpression is relevant. This inhibition of the cell cycle was consistent with the results of BrdU incorporation assays performed in chimeric mice, where we observed a reduction in BrdU-positive cells from 33±6% (control) to 17±1% (G0S2-overexpressing) in primitive hematopoietic progenitor cells (not shown). Conversely, G0S2 silencing in HSCs led to increased percentages of cells in the G_1_ and S phases of the cell cycle (Figure 4C). In addition to HSC, retroviral overexpression and silencing showed that G0S2 can also modulate proliferation in LS^−^K cells, a population enriched in hematopoietic progenitor cells (Figure S2). From these experiments, we concluded that G0S2 regulates the proliferation of hematopoietic stem and progenitor cells.

### G0S2 interacts with nucleolin and ribonucleoproteins

**Figure 5:**
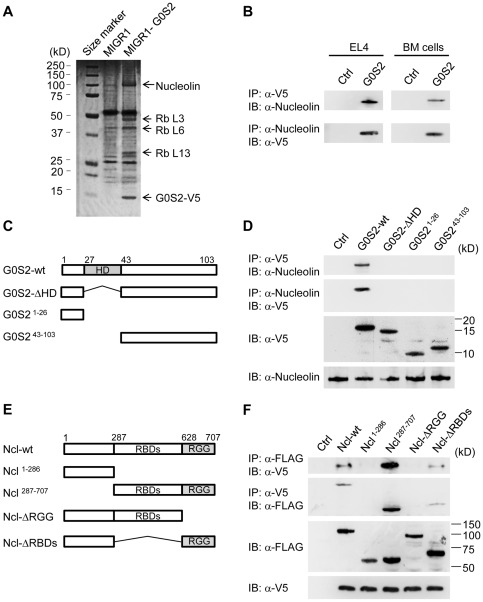
The hydrophobic domain of G0S2 interacts with the RGG domain of nucleolin. Co-immunoprecipitation and proteomic analyses were used to identify G0S2 protein partners. (A) G0S2-interacting proteins were pulled down with anti-V5 antibody in EL4 cells transduced with the empty or G0S2-V5 tagged retrovirus. Bands from a SDS-PAGE gel stained with Coomassie Blue were cut out for identification by mass spectrometry. (B) Reciprocal co-immunoprecipitation of V5-tagged G0S2 and nucleolin in EL4 and BM cells transduced with the MIGR1 (Ctrl) or MIGR1-G0S2 (G0S2) retrovirus. (C) Diagram depicting the domains in the wild-type G0S2 protein and the deletion mutants G0S2-ΔHD, G0S2^1–26^ and G0S2^43–103^. (D) The interaction between endogenous nucleolin and ectopic V5-tagged G0S2 was analyzed in NIH3T3 cells transduced with the empty retrovirus (Ctrl) or retroviruses bearing wild-type G0S2 (G0S2-wt) or G0S2 mutant constructs (G0S2-ΔHD, G0S2^1–26^ and G0S2^43–103^). (E) Diagram depicting the domains of the wild-type nucleolin (Ncl) protein and the deletion mutants Ncl^1–286^, Ncl^287–707^, Ncl-ΔRGG and Ncl-ΔRBDs (FLAG-tagged). RGG, arginine-glycine-glycine-rich domain; RBD, RNA-binding domain. (F) Interactions between V5-tagged G0S2 and FLAG-tagged nucleolin constructs (Ncl-wt, Ncl^1–286^, Ncl^287–707^, Ncl-ΔRGG and Ncl-ΔRBDs). The data represent two independent experiments.

Because G0S2 is a cytosolic protein with an unknown function, we hypothesized that G0S2 may interact with proteins involved in the control of cell division. To identify G0S2-interacting proteins in hematopoietic cells, we transduced murine lymphoma EL4 cells with the V5-tagged G0S2 retrovirus because the scarce number of HSCs precluded a proteomic approach. First, we confirmed that forced expression of G0S2 in EL4 cells reduced the percentage of S-phase cells relative to the proportion observed in cells transduced with an empty retrovirus (Figure S3). Next, cell lysates were immunoprecipitated with an anti-V5 antibody, and immune complexes were analyzed by sodium dodecyl sulfate polyacrylamide gel electrophoresis (SDS-PAGE). Proteins immunoprecipitated with anti-V5 in the lysate of V5-tagged G0S2-expressing cells but not control EL4 cells were excised from the gel and digested with trypsin for peptide mass fingerprinting using mass spectrometry. Four proteins with apparent molecular masses of 100, 47, 40, and 30 kDa were identified as nucleolin and ribosomal proteins L3, L6 and L13, respectively (Figure 5A). We focused on the nucleolin interaction because of its known roles in ribosomal RNA synthesis, chromatin remodeling, gene expression, and cell proliferation [22]. We confirmed the interaction between G0S2 and nucleolin in EL4 and BM cells by reciprocal co-immunoprecipitation using lysates from cells transduced with V5-tagged G0S2. We demonstrated that the anti-V5 antibody co-immunoprecipitated endogenous nucleolin and that the immunoprecipitation of endogenous nucleolin resulted in the reciprocal immunoprecipitation of V5-tagged G0S2 in lymphoblastic EL4 cells and, more importantly, in murine BM cells (Figure 5B).

We next determined the domains responsible for the interaction between G0S2 and nucleolin. G0S2 is a small, basic protein with a central and highly conserved hydrophobic domain (HD) flanked by N- and C-terminal domains (Figure 5C). We generated retroviruses carrying mutants with deletions of the hydrophobic domain (G0S2-ΔHD), the C-terminal and HD domains (G0S2^1–26^) or the N-terminal and HD domains (G0S2^43–103^). Full-length G0S2 interacted with nucleolin in NIH-3T3 cells, whereas the G0S2^1–26^, G0S2^43–103^ and G0S2-ΔHD mutants did not (Figure 5D). We then generated nucleolin mutants to determine the domains required for G0S2 binding. The nucleolin (Ncl) protein contains a cluster of 4 RNA-binding domains (RBDs) and an arginine-glycine-glycine (RGG)-rich domain that binds ribonucleoproteins. We generated the following nucleolin deletion mutants (Figure 5E): Ncl^1–286^ (the N-terminal domain, including the nuclear localization signal), Ncl^287–707^ (the C-terminal domain, containing both the RBD and RGG-rich domains), Ncl-ΔRGG (deletion of the RGG domain), and Ncl-ΔRBDs (deletion of all of the RBD domains). NIH3T3 cells were co-transfected with an expression vector containing a mutant or full-length nucleolin protein and the V5-tagged G0S2 retroviral construct. Co-immunoprecipitation assays showed that G0S2 only interacted with the nucleolin mutants that contained the RGG domain: wild type (Ncl-wt), Ncl^287–707^, and Ncl-ΔRBDs (Figure 5F). From these studies, we concluded that the hydrophobic domain of G0S2 binds to the RGG domain of nucleolin.

### The interaction between G0S2 and nucleolin leads to cytosolic retention of nucleolin and cell cycle inhibition in HSCs

**Figure 6:**
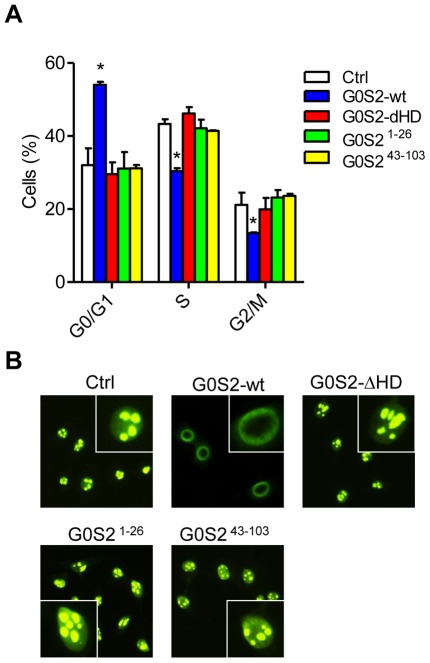
Inhibition of cell proliferation by G0S2 correlates with cytosolic retention of nucleolin. (A) The effects of G0S2 deletion mutants on the proliferation of NIH-3T3 cells were examined by propidium iodine staining and flow cytometric analysis (*n* = 3). G0S2-wt was compared to G0S2 mutants and empty vector (Ctrl). *, *P*<0.05 (two-tailed Student's *t*-test). (B) Subcellular localization of endogenous nucleolin was determined by immunofluorescence in NIH3T3 cells transduced with the retrovirus containing full-length G0S2 or the deletion mutants described in Figure 5. The inset shows a higher magnification of a cell presenting nucleolar versus perinuclear localization. The data represent two independent experiments.

We hypothesized that the interaction between G0S2 and nucleolin might affect the proliferation of hematopoietic cells. We transfected NIH3T3 cells with full-length or G0S2 mutants and then measured the cell cycle distribution. Expression of wild-type G0S2 reduced the percentage of cells in S phase; in contrast, the DNA contents of the G0S2 mutants that failed to interact with nucleolin were similar to those of the controls (Figure 6A). Intriguingly, G0S2-overexpressing cells showed a perinuclear distribution of nucleolin rather than the typical nucleolar localization observed in control cells (Figure 6B). Conversely, cells transfected with the G0S2-ΔHD, G0S2^1–26^ or G0S2^43–103^ construct showed a nucleolar localization of nucleolin, likely due to the inability of the mutant proteins to interact with nucleolin via the HD domain (Figure 6B).

**Figure 7:**
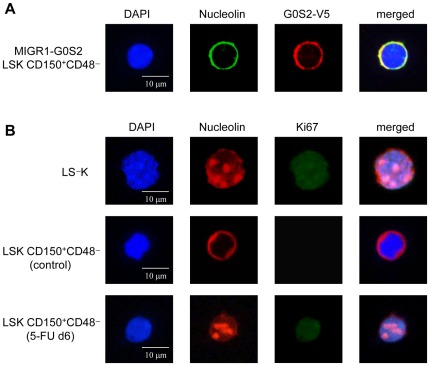
Quiescent HSCs exhibit cytosolic sequestration of nucleolin. (A) Nucleolin colocalizes with the overexpressed G0S2 protein in LSK CD150^+^ CD48^−^ cells purified from mice transplanted with BM cells transduced with the MIGR1-G0S2 V5-tagged retrovirus. (B) Expression of nucleolin and Ki67 was determined in wild-type LS^−^K (proliferative progenitors), LSK CD150^+^ CD48^−^ cells purified from wild-type mice (dormant HSCs), and LSK CD150^+^ CD48^−^ cells purified from wild-type mice injected 6 days earlier with a single dose of 5-FU (proliferative HSCs). Images of DAPI, nucleolin, and Ki67 staining are shown for a representative cell. The data represent two independent experiments.

The proteomic identification of G0S2 protein partners was performed using EL4 cells, whereas the molecular interaction assays were performed using fibroblasts. To correlate these findings with those related to HSC proliferation, we examined whether the G0S2-nucleolin interaction occurs in quiescent but not proliferating HSCs. We first examined the localization of nucleolin in HSCs purified from mice transplanted with MIGR1-G0S2-V5 BM cells. Similar to the findings in cell lines, endogenous nucleolin co-localized with the overexpressed G0S2-V5 tagged protein around nuclei in LSK CD150^+^ CD48^−^ cells (Figure 7A). Because the majority of LSK CD150^+^ CD48^−^ cells are in G_0_, this perinuclear distribution of nucleolin in G0S2-overexpressing HSCs suggests that cytosolic retention of nucleolin might cause a reduction in stem cell proliferation. Remarkably, endogenous nucleolin presented the same ring-like distribution in wild-type LSK CD150^+^ CD48^−^ cells, whereas it was restricted to the nucleoli in Ki67^+^ LS^−^K progenitor cells (Figures 7B and S3). We next isolated LSK CD150^+^ CD48^−^ cells from mice injected 6 days before with 5-FU to induce cytoablation and regenerative HSC proliferation. In proliferating HSCs, nucleolin was predominantly localized to the nucleoli, similar to proliferating progenitor cells (Figures 7B and S3). Nucleolar localization in cycling stem and progenitor cells correlated with low G0S2 levels (Figure 1C). Collectively, our data show that increased levels of G0S2 in nonproliferative HSCs induce cytosolic retention of nucleolin, preventing nuclear shuttling and regulation of cell division.

## Discussion

The functional longevity of HSCs relies on their ability to balance proliferation, differentiation, and quiescence. Stem-cell intrinsic activators of quiescence can prevent the loss of stemness by modulating responses to differentiation-inducing signals. In this study, we found that G0S2 restricts the steady-state number of HSCs entering the cell cycle in a cell-autonomous manner. Furthermore, we showed that elevated levels of G0S2 in HSCs cause retention of nucleolin in the cytosol and inhibition of cell proliferation.

The initial finding that G0S2 expression is inversely correlated with cyclin E2 levels in LSK CD150^+^ CD48^−^ cells, a population highly enriched for HSCs [27], [28], [29], [30], suggested that G0S2 may be involved in the maintenance of stem cell quiescence. Competitive BM transplants revealed that HSCs overexpressing G0S2 were able to contribute to the hematopoietic system over the long term, although with lower efficiency than wild-type HSCs. Conversely, retroviral silencing of the endogenous G0S2 gene in BM cells resulted in increased contributions to the peripheral myeloid and lymphoid cell populations upon transplantation. We hypothesized that G0S2 primarily regulates the decision to proliferate or remain quiescent in HSCs: ectopic G0S2 expression led to an increased percentage of quiescent HSCs, whereas silencing resulted in increased HSC proliferation. We also observed that G0S2 modulates at some extent the proliferation of hematopoietic progenitor cells although myeloid committed progenitor cells appear to be less dependent on G0S2 regulation. Consistent with increased homing of quiescent HSCs [32], ectopic G0S2 expression led to increased chimerism in the BM but lowered the contribution to blood in competitive transplants. This result is more likely attributable to reduced HSC proliferation than decreased differentiation because no differences were observed in the number of myeloid colonies in clonogenic assays. In support of our findings, a gene expression analysis in fibroblasts subjected to prolonged quiescence revealed that upregulation of G0S2 was part of a gene signature that suppresses proliferation [33].

Other factors known to induce HSC quiescence are p21, Gfi1, Pten, and FoxO1, 3 and 4 [34], [35], [36], [37]. Knockout mice deficient in these factors showed increased homeostatic cycling of HSCs and eventual stem cell exhaustion, although augmented HSC proliferation does not always cause a loss of stem cell function [38], [39], [40]. For instance, overexpression of HoxB4 uncouples cell division from differentiation in HSCs, leading to *ex vivo* HSC expansion [39]. Our data suggest that modulations of G0S2 expression might be applied to preserve HSC stemness during *ex vivo* culture, without the side effect of promoting differentiating cell division events that might compromise long-term stem cell function. However, the upstream signaling pathways and transcription factors that regulate G0S2 expression in HSCs are largely unknown. The only activator of G0S2 described in hematopoietic cells is retinoic acid [41]. Interestingly, retinoic acid treatment enhances *ex vivo* maintenance of HSCs [42]. Similar to G0S2, Stat5 activity promotes HSC quiescence [43], raising the possibility that G0S2 could be regulated downstream of c-kit and Mpl signaling. However, inhibition of calcium-dependent calcineurin with cyclosporin A suppresses G0S2 transcription in human blood mononuclear cells [7], and deletion of calmodulin-dependent protein kinase IV resulted in hematopoietic failure due to a numeric and functional reduction of HSCs [44]. Future studies are required to define the regulation of G0S2 expression in HSCs.

The cytosolic localization of G0S2 suggested that the G0S2-mediated inhibition of HSC proliferation likely comprises a non-transcriptional mechanism involving protein-protein interactions. In a proteomic analysis, we identified nucleolin as a new protein partner of G0S2 and further showed that the hydrophobic domain of G0S2 is required for this interaction. Nucleolin is a phosphonucleolar protein involved in several phases of ribosome biosynthesis: transcription of ribosomal DNA in nucleoli, maturation of pre-ribosomal RNA, and transport of ribonucleoproteins and pre-ribosomal particles for ribosome assembly of ribosomes in the cytosol [22], [45], [46]. However, the role of nucleolin in the HSC proliferation has not been well studied, although nucleolin's function is generally thought to be associated to cell growth and cell division. Nucleolin expression is elevated in rapidly dividing cells and tumor cells, supporting a role for this protein in cell proliferation [26]. Conversely, silencing of nucleolin expression in HeLa cells and human fibroblasts reduced both the percentage of cells in S phase and cell growth [47]. In hematopoietic cells, retinoblastoma protein antagonizes the nucleolin-mediated activation of the CD34 promoter in KG1 acute myelogenous leukemia cells [48]. Finally, nucleolin may contribute to the IFNα-mediated release of HSCs from quiescence due to its ability to transport Stat1 proteins into the nucleus [49], [50]. Therefore, G0S2 might modulate proliferation by interfering with the pro-proliferation functions of nucleolin. In addition to nucleolin, we also determined that ribonucleoproteins L3, L6, and L13 bind to G0S2. Several ribosomal proteins, including L3, L6 and L13, interact with nucleolin via the RGG domain in the C-terminal moiety [51]. The fact that nucleolin also binds to the ribonucleoproteins found in our screen of G0S2-interacting proteins indicates that G0S2 either binds directly to the RGG domain or to a complex containing ribonucleoproteins and nucleolin.

Our overexpression study suggests that G0S2 interacts with nucleolin when its cellular level reaches a certain threshold. This interaction leads to the retention of nucleolin in the cytosol and suppression of cell division. In support of the physiological relevance of our findings, we demonstrated that wild-type LSK CD150^+^ CD48^−^ cells, a population with high levels of endogenous G0S2 gene expression, displayed a perinuclear distribution of nucleolin. In contrast to the predominantly ‘ring-like’ distribution observed in HSCs, progenitor cells (LS^−^K) showed both perinuclear and nucleolar localization of nucleolin, suggesting a lower dependency on the G0S2 and Nucleolin interaction. This difference might be related to the levels of G0S2 expression in stem versus progenitor cells. The ring-like extranuclear distribution of nucleolin was previously described in nonproliferating leukemic cells of chronic lymphocytic leukemia patients [24]. Similarly, T lymphocytes from HIV-positive patients show a cytosolic expression of nucleolin that is not observed in healthy donors [52]; however, this subcellular localization is associated with apoptosis in CD4 T cells. Our work is the first report to describe a mechanism for the subcellular redistribution of nucleolin and the association of G0S2 with proliferation in hematopoietic cells.

Most research in the field has focused on identifying the transcriptional machinery that controls stem cell proliferation. Our work supports a novel role for G0S2 in the regulation of nucleolin function by its sequestration of nucleolin in the cytosol in quiescent HSCs. This mechanism could potentially be targeted to reduce cell-cycle-dependent cytotoxicity and improve engraftment in bone marrow transplants.

## Material and Methods

### Mice

C57BL/6 (CD45.2) and B6.SJL (CD45.1) mice were purchased from Jackson Laboratories. All mice were bred and maintained under specific pathogen-free conditions at the Baylor College of Medicine. Chemical cytoablation was used to study hematological recovery by intraperitoneal administration of a single dose of 5-fluorouracil (5-FU) at a dose of 150 mg/kg. All experiments were performed with the approval of the Institutional Animal Care and Usage Committee of Baylor College of Medicine.

### Cell lines

NIH3T3 and EL4 cells were obtained from the ATCC. NIH3T3 cells were cultured in DMEM (Lonza) containing 10% (vol/vol) FBS. EL4 cells were cultured in RPMI medium (Lonza) containing 10% (vol/vol) FBS.

### Retroviral transduction

Murine G0S2-V5 tagged cDNA was cloned into the MIGR1 retroviral vector [53]. The luciferase-specific shRNA retroviral construct (pSIREN-Luc) was purchased from Clontech and two G0S2-specific shRNA (pSIREN-sh1 and pSIREN-sh2) retroviral vectors were generated to induce G0S2 gene silencing. The G0S2 shRNA sequences were as follows: mouse sh1, 5′-CGAGCAATCAA GGAGCTAT-3′; mouse sh2, 5′-GAGTCACATGCTGTTTCAA-3′. HEK 293T cells were cotransfected with a plasmid containing the retroviral vectorand ψ–ecotropic envelope. After 2 days of transfection, supernatant containing retrovirus was passed through a 0.4 µm filter before transduction. BM cells were flushed out from the femur and tibias of mice previously treated with 5-FU (150 mg/Kg) and cultured for two days in the presence of stem cell factor (100 ng/ml, Peprotech), IL-3 (6 ng/ml, Petrotech) and IL-6 (10 ng/ml, Peprotech) in X-vivo 15 medium (Lonza). Cells were then transduced twice by spinoculation (60 min at 456 RCF) in the presence of polybrene (8 µg/ml). Next, 1×10^6^ BM cells were injected intravenously into the lateral tail vein of recipient mice that were previously irradiated with 950 Rad. NIH3T3 cells were transduced with retroviral supernatant in the presence of 8 µg/ml polybrene. EL4 cells were cocultured with packaging 293 T cells for 2 days, followed by purification of EGFP-positive, nonadherent cells by cell sorting.

### Flow cytometry and cell purification

BM cells were flushed out from their tibias and femurs, and hematopoietic progenitors were purified by lineage depletion using the BD-IMag magnetic-bead separation system (BD Biosciences) following the manufacturer's instructions. Splenocytes were filtered through 40 µm nylon cell strainer (BD Biosciences) to obtain single-suspension cells. Blood samples were treated with a hypotonic reagent for a lysis of red blood cells before staining. The following antibodies were used to stain peripheral blood and BM cells with the retroviral transduction: PE-Cy7-anti-CD11b (BD Biosciences), APC-anti-Gr-1 (BD Biosciences), APC-anti-CD3(BD Biosciences), PE-Cy7-anti-CD19 (BD Biosciences), APC-anti-CD45.1 (eBioscience). Samples were analyzed by flow cytometry using a FACSCanto instrument (BD Biosciences), and the resultant data were analyzed using FlowJo software (Tree Star).

For isolation of LSK CD150^+^ CD48^−^ CD41^−^ and LSK CD150^+^ CD48^−^ HSCs, LSK CD150^−^ CD48^−^ CD41^−^, FcγRII/III^lo^ CD34^+^, FcγRII/III^hi^ CD34^+^, FcγRII/III^−^ CD34^−^, CD3^+^ CD4^+^, CD3^+^ CD8^+^, and CD19^+^ cells, we used the following antibodies: PE- or APC-anti-CD150 (BioLegend), FITC- or anti-CD48 (BioLegend) or PE-Cy7-anti-CD48 (eBioscience), FITC-anti-CD41 (BD Biosciences), PerCP/Cy5.5-anti-Sca-1 (BD Biosciences) or FITC-Sca-1(BioLegend), APC-anti-c-kit (BD Biosciences) or PE-Cy7-anti-c-kit (BioLegend), FITC-anti-CD16/32 ( = FcγRII/III) (BioLegend), APC-anti-CD34 (BioLegend), PE-anti-CD11b (BD Biosciences), APC-anti-Gr-1(BD Biosciences), APC-anti-CD3(BD Biosciences), FITC-anti-CD4 (BD Biosciences), PE-anti-CD8 (BD Biosciences), PE-anti-CD19 (BD Biosciences). Cells were sorted using a MoFlo cell sorter (Cytomation).

### Cell cycle analysis

Purified EGFP-positive BM cells and NIH3T3 cells were centrifuged and resuspended in a hypotonic buffer (0.1% sodium citrate and 0.1% Triton X-100) containing 100 µg/ml RNase A and 50 µg/ml propidium iodide. Samples were analyzed using the FACScanto flow cytometer (BD Biosciences), and cellular DNA content were analyzed using the ModFit software (Verity). Purified EGFP-positive or ZsGreen-positive Lin^−^ Sca-1^+^ c-kit^+^ CD150^+^ CD48^−^ (LSK CD150^+^ CD48^−^) HSCs and Lin^−^ Sca-1^−^ c-kit^+^ progenitors from BM were fixed in 70% ethanol for 16 h and stained with APC-anti-Ki67 (eBioscience) and 7-AAD (BD Biosciences). Samples were analyzed using the FACScanto flow cytometer and FlowJo software (Tree Star).

### Real-time quantitative PCR

Total RNA was extracted using the RNeasy Mini kit or RNeasy Plus Micro kit (Qiagen), and cDNA was then synthesized from RNA using the SuperScript III kit (Invitrogen) with random hexamer primers. Real-time PCR was performed using the LightCycler FastStart DNA Master SYBR Green I (Roche). Primer sequences for PCR were as follows: mouse β-actin forward, 5′-GTGGGCCGCTCTAGGCACCA-3′, and reverse, 5′-CGGTTGGCCTTAG GGTTCAGGGG-3′; mouse G0S2 forward, 5′-GTGCTCGGCCTAGTTGAGAC-3′, and reverse, 5′-CACCTGGGTCATGATCTGTG-3′; mouse Cyclin E2 forward, 5′-AGGAATCAGCCCTTGCAT TATC-3′, and reverse, 5′-CCCAGCTTAAATCTGGCAGAG-3′. Real-time PCR was performed using the Mx3005P instrument (Stratagene, La Jolla, CA) with denaturation at 95°C for 10 min followed by 40 cycles of a 3-step PCR program consisting of 95°C for 15 sec, 55°C for 30 sec and 72°C for 30 sec. Gene expression was normalized to β-actin levels (1×10^4^ copies).

### Co-immunoprecipitation and immunoblot

EL4 cells, BM cells and NIH3T3 cells were transduced with retroviruses, MIGR1 or MIGR1-G0S2. NIH3T3 cells were cotransfected with plasmids for V5-tagged G0S2 and FLAG-tagged nucleolin expression using Lipofectamine 2000 (Invitrogen). After 48 h, cells were lysed with a solution of 150 mM NaCl, 50 mM Tris pH 8, and 1% Triton X-100 containing a protease inhibitor cocktail (Calbiochem). Cell lysate was immunoprecipitated with Protein G-sepharose (Invitrogen) and mouse anti-V5 antibody (Invitrogen), rabbit anti-Nucleolin antibody (Abcam) or rabbit anti-FLAG antibody (Cell Signaling technology). The samples were then loaded onto NuPAGE Bis-Tris gels (Invitrogen), separated by electrophoresis, and transferred onto polyvinylidene difluoride membranes (Millipore) for immunoblot analysis. Goat anti-rabbit IgG or goat anti-mouse IgG conjugated to peroxidase (GE Healthcare) was used as a secondary antibody. The chemiluminescent signals were gained with SuperSignal substrate (Thermo Scientific). The bands were analyzed using the FluorChemHD2 Chemi-Imager (Alpha Innotech, Santa Clara, CA).

### Mass spectrometry analysis

EL4 cells were transduced with retroviruses and lysed as described above for co-immunoprecipitation. Cell lysates were immunoprecipitated with Protein G-sepharose and anti-V5 antibody (Invitrogen). The immunoprecipitated proteins were then resolved on NuPAGE Bis-Tris gels (Invitrogen) and stained with Coomassie Blue R-250 (Fluka). Stained gel bands were dehydrated and trypsinized, and the peptides were then analyzed using an ABI/SCIEX 4700 Proteomic Analyzer TOF/TOF mass spectrometer (The Proteomic Core Facility at Baylor College of Medicine). Peptide mass fingerprinting was then analyzed using MS-Fit and protein database searches.

### Methylcellulose culture

BM cells were transduced with retroviral vectors (MIGR1 or MIGR1-G0S2) and transplanted into irradiated mice. After two months of hematologic reconstitution, 2×10^4^ EGFP^+^ BM cells were cultured in 6-well plates with methylcellulose medium (MethoCult GFM3434, StemCell Technologies) to enumerate myelopoietic progenitor cells. After 9–10 days, colonies were enumerated, and each well was then resuspended for total cell count. For serial replating, isolated 2×10^4^ cells were replated on methylcellulose.

### Generation of deletion mutants

Full-length murine G0S2 cDNA was cloned into the MIGR1 retroviral vector MIGR1 with a V5-tag and pcDNA3.1/V5-His A (Invitrogen). G0S2 deletion mutants were generated by PCR using the following primers, designed to generate deletion mutants G0S2^1–26^ (N-terminal) and G0S2^43–103^ (C-terminal), respectively: G0S2-N forward, 5′-ATGGAAAGTGTGCAG-3′; G0S2-N reverse, 5′-TAGCTTCACTAGCTTCC-3′; G0S2-C forward, 5′-GCCACCATGGTTGAGACGGTGTGCAG-3′; and G0S2-C reverse, 5′-AGAGGCGTGCTGCCGGA-3′. For the G0S2-ΔHD, G0S2^1–26^ and G0S2^43–103^ mutants, PCR products were combined using the following primers: G0S2-N/C sense, 5′-AAGCTAGTGAAGCTAGTTG AGACGGTGTGC-3′, and G0S2-N/C antisense, 5′-GCACACCGTCTCAACTAGCTTCACTAGCTT-3′. The full-length cDNA of murine nucleolin was cloned into the p3XFLAG-Myc-CMV-24 expression vector (Sigma). Nucleolin (Ncl) deletion mutants were generated by PCR amplification using the following primers, designed to generate the deletion mutants Ncl^1–286^, Ncl^287–707^, Ncl-ΔRGG, respectively: Ncl-N forward, 5′-TTTGAATTCTATGGTGAAGCTCGCAAAG-3′; Ncl-N reverse, 5′- TTTGAATTCTCTCCTTCTTCCGTTTTCCAG-3′; Ncl-C forward, 5′- TTTGAATTCTATGACCAAGCAGAAAGAAG-3′; Ncl-C reverse, 5′- TTTGAATTCTTTCAAACTTCGTCTTCTTTCC-3′; Ncl-ΔRGG forward, 5′- TTTGAATTCTATGGTGAAGCTCGCAAAG-3′; and Ncl-ΔRGG reverse, 5′-TTTGAATTCTGGCCTCCTTGGCAGCTTTG-3′. For the Ncl-ΔRBD mutants, the Ncl^1–286^ and Ncl^627–707^ fragments were generated first using the following primers: Ncl-N′ forward, 5′-TTTGAATTCTATGGTGAAGCTCGCAAAG-3′; Ncl-N′ reverse, 5′- GTCAATTTCTCCATCTTCCATCTCCTTCTTCCGTTTTCCAG-3′; Ncl-RGG forward, 5′- CTGGAAAACGGAAGAAGGAGATGGAAGATGGAGAAATTGAC-3′; Ncl-RGG reverse, 5′- TTTGAATTCTTTCAAACTTCGTCTTCTTTCC-3′. Finally, Ncl^1–286^ and Ncl^627–707^ were combined by using the primers Ncl-N′ forward and Ncl-RGG reverse.

### Immunofluorescence detection

LSK CD150^+^ CD48^−^ HSCs and lineage^−^ Sca-1^−^ c-kit^+^ (LS^−^K) progenitors were purified by cell sorting of BM cells collected from non-transplanted or transplanted mice two months prior with transduced MIGR1 or MIGR1-G0S2 BM cells. Suspension cells were spun onto glass slides (Thermo) and immediately fixed with 70% ethanol, a condition that abolished prestained fluorescence. NIH3T3 cells were cultured in 8-well culture slides (BD Biosciences) and fixed with 1% paraformaldehyde and permiabilized with 0.1% (vol/vol) Triton X-100. The slides were stained with mouse anti-V5 and mouse-specific antibody conjugated to Alexa Fluor 555 (Invitrogen). Subcellular colocalization was determined using an antibody kit (Cell Signaling Technology), anti-COX IV (Abcam), anti-calnexin (Abcam), and a rabbit-specific antibody conjugated to Alexa Fluor 488. Nucleolin was detected by rabbit anti-nucleolin (Abcam) and a rabbit-specific antibody conjugated to Alexa Fluor 488 or Alexa Fluor 555 (Invitrogen). Ki67 was stained with FITC-anti-Ki67 (Vector Laboratories). The slides were mounted with a mounting solution containing 4′-6-diamidino-2-phenylindole (DAPI) (Invitrogen) and analyzed on the microscope Eclipse 90i (Nikon) and the imaging software NIS Elements (Nikon).

### Bone marrow homing assay

BM cells were harvested from 5-FU treated mice and transduced with either MIGR1-G0S2 or MIGR1 empty retrovirus. Twenty-four hours later, EGFP-positive cells were purified by cell sorting and injected (1×10^5^ cells) into the tail veins of irradiated recipient mice. BM cells were isolated from the mouse femurs 15 h later to determine the percentage of EGFP-positive cells by flow cytometry.

### Statistical analysis

Significant differences were determined using a two-tailed Student's t-test (GraphPad solftware). A p value of <0.05 was considered significant. Statistics are indicated in each figure legend.

## Supporting Information

Figure S1 **Ectopic expression of G0S2 enhances homing of BM cells without inducing apoptosis.** (A) The profile of Annexin V and 7-AAD is shown for gated EGFP+ BM cells transduced with MIGR1 or MIGR1-G0S2 retrovirus (*n* = 3). (B) Engraftment assay of BM cells transduced with either MIGR1 or MIGR-G0S2 retrovirus was analyzed after 15 h of i.v. injection (*n* = 3). *, *P*<0.05 (two-tailed Student's *t*-test).
(TIF)

Figure S2 **Cell cycle analysis of progenitor cells.** LS^−^K cells were sorted from BM transplanted with MIGR1-G0S2 (A) or pSIREN-shG0S2 (B), fixed in 70% ethanol, and stained with Ki67 and 7-AAD (*n* = 3–5). *, *P*<0.05, and **, *P*<0.01 (two-tailed Student's *t*-test).
(TIF)

Figure S3 **Ectopic expression of G0S2 inhibits proliferation in EL4 cells and induced perinuclear sequestration of nucleolin in resting HSCs.** (A) Cell growth was analyzed in EL4 cells transduced with retroviruses MIGR1 or MIGR1-G0S2 (*n* = 3). **, *P*<0.01 (two-tailed Student's *t*-test). (B) Cell cycle analysis by DNA staining with propidium iodine. (C) LSK CD150^+^CD48^−^ BM cells isolated from B6 and 5-FU-injected B6 mice. Localization of nucleolin was determined by immunofluorescence.
(TIF)
