# Supplementary figures and images for: The Cytosolic Protein G0S2 Maintains Quiescence in Hematopoietic Stem Cells
Source: PLoS One. 2012 May 31;7(5):e38280. doi: 10.1371/journal.pone.0038280 (PMC3365016; doi:10.1371/journal.pone.0038280)

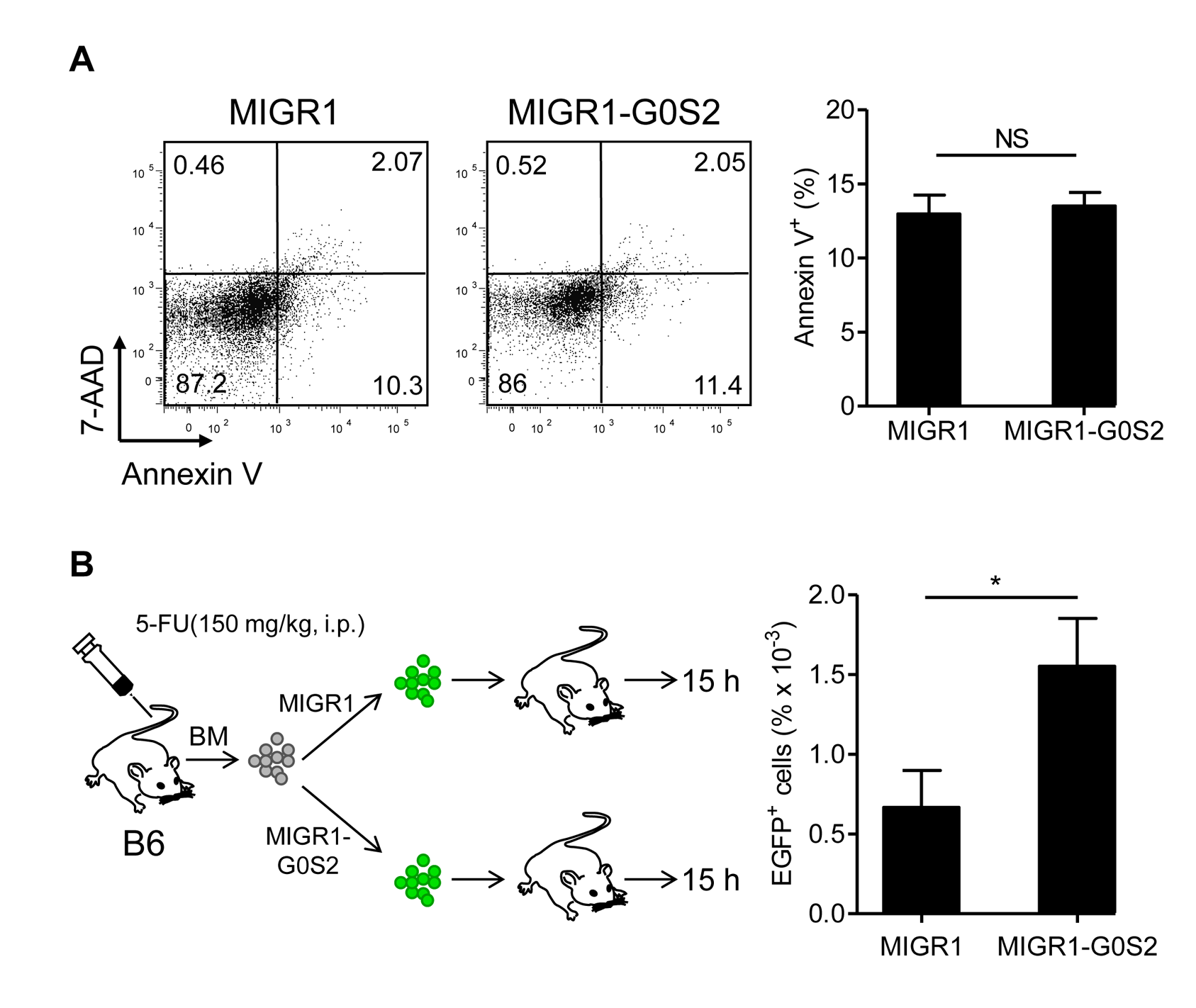

Supplement: Figure S1 — Ectopic expression of G0S2 enhances homing of BM cells without inducing apoptosis. (A) The profile of Annexin V and 7-AAD is shown for gated EGFP+ BM cells transduced with MIGR1 or MIGR1-G0S2 retrovirus (n = 3). (B) Engraftment assay of BM cells transduced with either MIGR1 or MIGR-G0S2 retrovirus was analyzed after 15 h of i.v. injection (n = 3). *, P<0.05 (two-tailed Student's t-test). (TIF) [file pone.0038280.s001.tif]

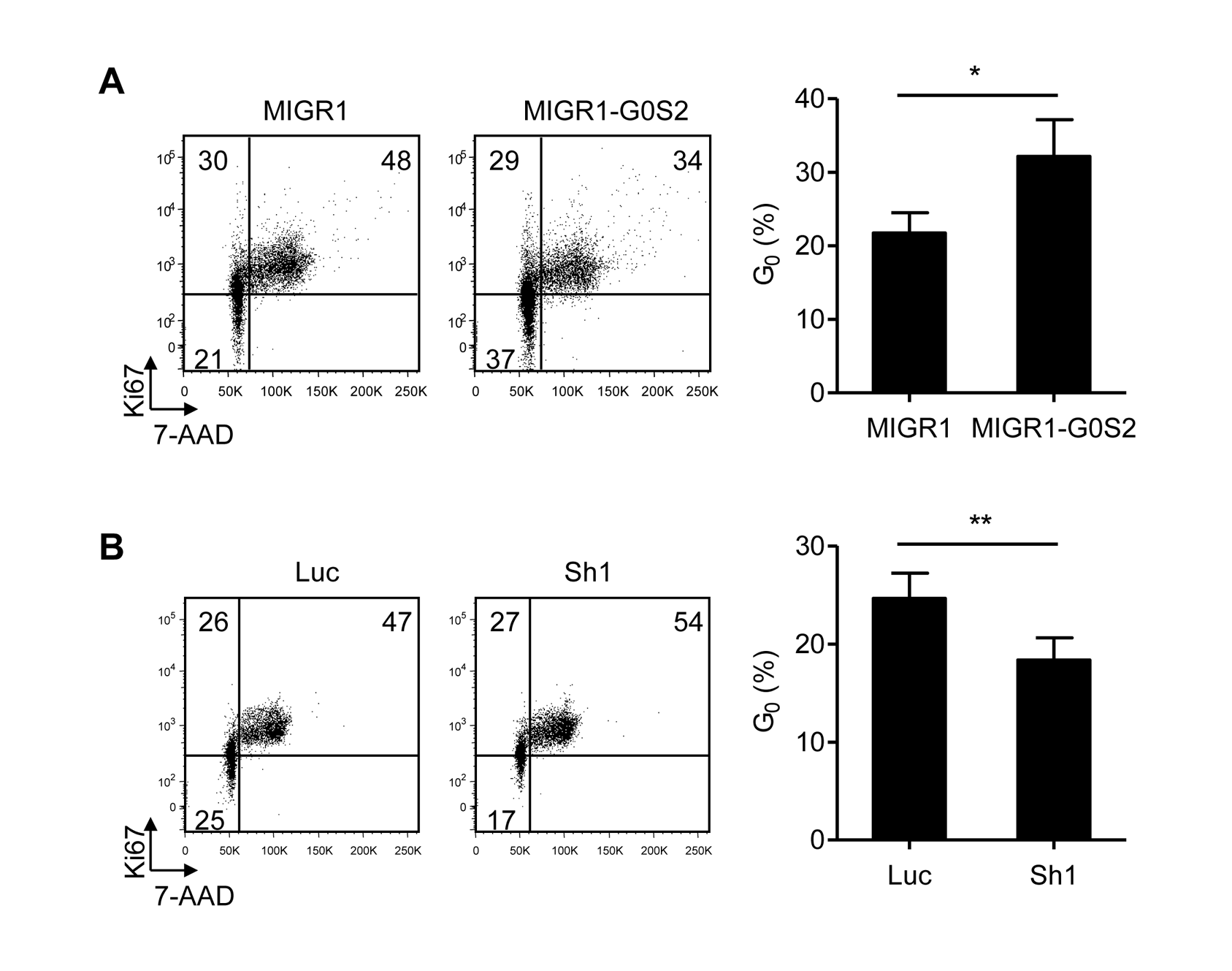

Supplement: Figure S2 — Cell cycle analysis of progenitor cells. LS−K cells were sorted from BM transplanted with MIGR1-G0S2 (A) or pSIREN-shG0S2 (B), fixed in 70% ethanol, and stained with Ki67 and 7-AAD (n = 3–5). *, P<0.05, and **, P<0.01 (two-tailed Student's t-test). (TIF) [file pone.0038280.s002.tif]

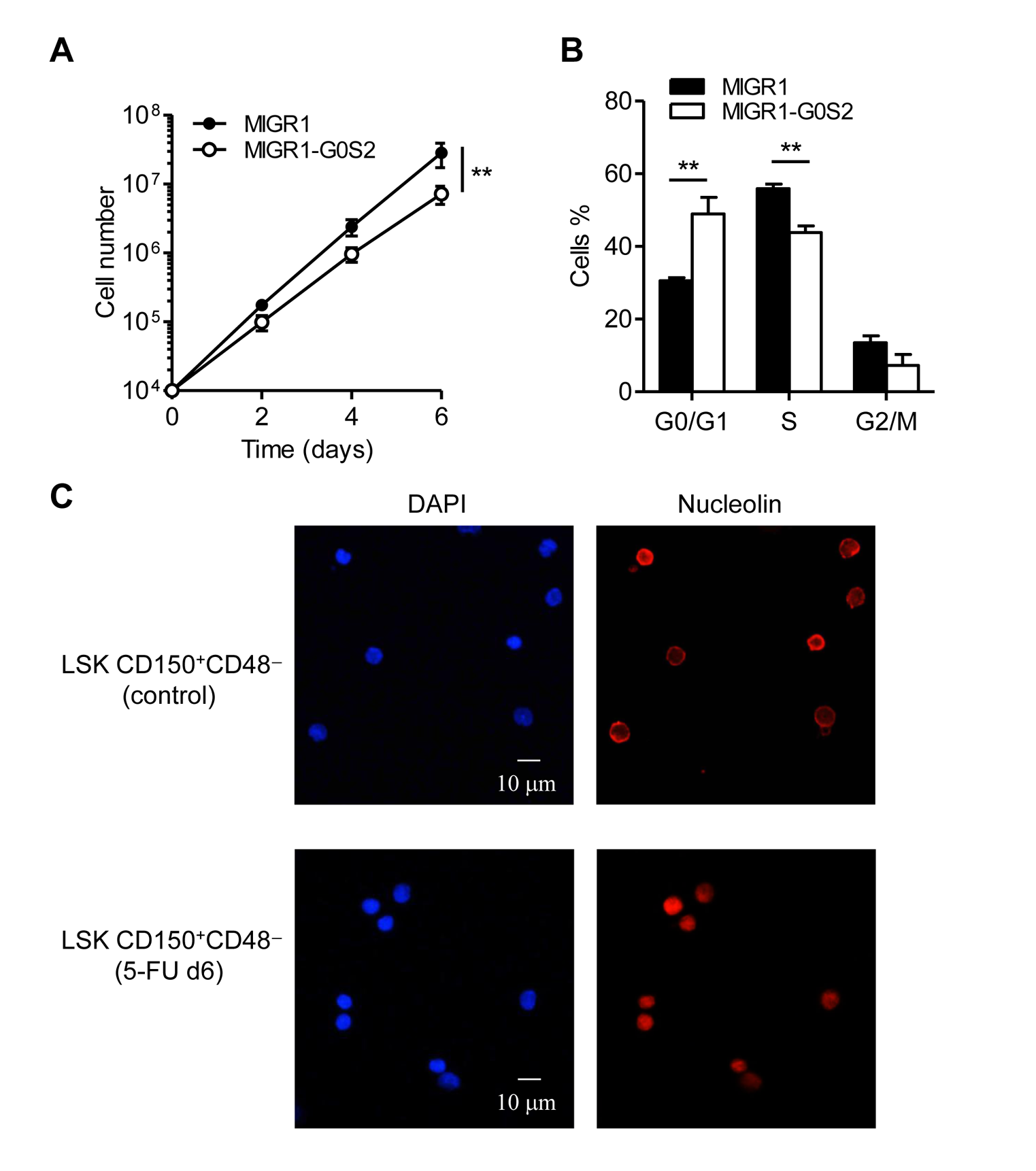

Supplement: Figure S3 — Ectopic expression of G0S2 inhibits proliferation in EL4 cells and induced perinuclear sequestration of nucleolin in resting HSCs. (A) Cell growth was analyzed in EL4 cells transduced with retroviruses MIGR1 or MIGR1-G0S2 (n = 3). **, P<0.01 (two-tailed Student's t-test). (B) Cell cycle analysis by DNA staining with propidium iodine. (C) LSK CD150+CD48− BM cells isolated from B6 and 5-FU-injected B6 mice. Localization of nucleolin was determined by immunofluorescence. (TIF) [file pone.0038280.s003.tif]
